# Efficient zinc ion biosorption on the biomass of *Ericaria selaginoides* algae following response surface methodology optimization: uptake behaviors and mechanisms

**DOI:** 10.7717/peerj.21793

**Published:** 2026-10-14

**Authors:** Mustafa A. Fawzy, Mona Alharbi, Hana Sonbol, Mohamed R. Fouad, Shanoo Sheikh, Sedky H.A. Hassan

**Affiliations:** 1 Botany and Microbiology Department, Faculty of Science, Assiut University, Assiut, Egypt; 2 Biology Department, College of Sciences, Taif University, Taif, Saudi Arabia; 3 Department of Biology, College of Science, Princess Nourah bint Abdulrahman University, Riyadh, Saudi Arabia; 4 Department of Pesticide Chemistry and Technology, Faculty of Agriculture, Alexandria University, El-Shatby, Alexandria, Egypt; 5 Department of Health Sciences, College of Health and Rehabilitation Sciences, Princess Nourah bint Abdulrahman University, Riyadh, Saudi Arabia; 6 Department of Biology, College of Science, Sultan Qaboos University, Muscat, Oman

**Keywords:** Removal, Zinc ions, Brown algae, Equilibrium isotherms, Thermodynamic, Response Surface Methodology

## Abstract

**Background:**

Biosorption using marine algae biosorbents is a sustainable and effective method for removing of heavy metals from aquatic environments.

**Methods:**

This study explored the biosorption of zinc ions from aqueous solutions using the biomass of the brown alga *Ericaria selaginoides*. The impacts of biosorbent dose, pH, and biosorbate concentration were optimized using the Response Surface Methodology (RSM) and 3-D surface plots generated via Design-Expert software.

**Results:**

Under optimized conditions, RSM predicted a maximum zinc removal efficiency of 89.79% which matches the experimental value of 87.72% ± 7.3%. These conditions included biosorbent dosage of 0.28 g/100 mL, zinc concentration of 33.62 mg/L, and pH of 6.97. The Langmuir model showed a maximum biosorption capacity of 114.94 mg/g, while the kinetic data were best described by the pseudo-second order model. The combined adsorption equilibrium, kinetic, thermodynamic, and surface characterization data show that Zn(II) biosorption by *E. selaginoides* involves a heterogeneous physicochemical process that includes surface complexation, ion exchange and auxiliary co-ordination with hydroxyl, amine, and phosphate groups. Overall, *E. selaginoides* exhibits promising potential as a low-cost and environmentally friendly biosorbent for zinc remediation from contaminated water, and additional process optimization could increase its efficacy in wastewater treatment. This research uniquely combines the optimization of zinc uptake by *E. selaginoides* using RSM with experimental evidence of the underlying biosorption behavior and mechanism.

## Introduction

Heavy metal pollution is currently a serious worldwide environmental problem, particularly in aquatic systems affected by mining, manufacturing, and other anthropogenic activities (Patel et al., 2026). Among these pollutants, zinc is a significant metal of environmental relevance because its high concentrations can adversely affect aquatic organisms and may pose risks to human health when exposure is prolonged (Olowoyo et al., 2026). Zinc exposure can result in typical symptoms such as nausea, irritability, and appetite loss in both humans and animals (Sharma, Sahoo & Pattnaik, 2026). Zinc contamination is associated with activities such as coal combustion, steel processing, and mining, and other industrial operations, which can introduce substantial zinc loads into wastewater and receiving water bodies.

According to the United States Environmental Protection Agency (USEPA), the secondary maximum contaminant level in drinking water should not contain more than five mg/L of zinc (USEPA, 2009). World Health Organization (WHO) reports that zinc concentrations in surface water and groundwater normally do not exceed approximately 0.01 and 0.05 mg/L, respectively, (WHO, 2011). These concerns highlight the need for efficient and environmentally sustainable approaches for Zn(II) removal from contaminated water.

Research into ways to eliminate these harmful contaminants from the environment due to water contamination with heavy metals has increased (Liu et al., 2026; Qader, Anwer & Ismael, 2025). Ion exchange, adsorption, precipitation, and membrane filtration are some of the advanced methods used to remove heavy metals; nevertheless, they are frequently expensive and may result in secondary contamination (Razzak et al., 2022; Zhang et al., 2020). In order to lower the high costs of water treatment and significantly reduce water pollution, researchers are constantly seeking ways to use affordable natural materials (Atif Irshad et al., 2026). In recent years, biosorption has emerged as an economical and environmentally friendly technique for eliminating heavy metals from wastewater (Jimoh, Azeez & Jamiu, 2026; Alnasrawy, 2026). The biosorption process involves complex interactions between metal ions and active binding sites on the biosorbent surface, including sulfate, carboxyl, hydroxyl, and amine groups (Carreira et al., 2023).

Microorganisms such as bacteria, fungi, yeast, and algae, as well as agricultural wastes such as leaves and root tissues, can be employed as biosorbents for detoxifying toxic or hazardous metals form industrial wastes (Kumar et al., 2026; Jimoh, Azeez & Jamiu, 2026). Marine brown algae are among the most promising bioabsorbents because of the density of carboxyl groups in the alginates, which constitute the basic constituent of their cell walls, their abundance and availability in seas and oceans, their high removal efficiency, high absorption capacity, and selective sensitivity to certain heavy metals (Salama et al., 2019; Kumar et al., 2015).

The brown algae most commonly investigated as biosorbents are *Padina gymnospora* (Bibak et al., 2025), *Cystoseira barbata* and *Hydroclathrus clathratus* (Ali, Kandil & Ibraheem, 2020), *Sargassum latifolium* (El-Sheekh et al., 2023; Moussa et al., 2022), and *Sargassum latifolium* and *Sargassum crassifolium* (Farhoud, Younis & Ali, 2024).

*Ericaria selaginoides*, a species of brown algae (Phaeophyta) found in the Red Sea coast of Jeddah, Saudi Arabia, has demonstrated promising potential as a biosorbent for heavy metal remediation, owing to its unique biochemical composition rich in polysaccharides, proteins, and phenolic compounds that facilitate metal binding (Kumar, Sidharth & Kandasubramanian, 2023; Periyasamy, Suresh Kumar & Subba Rao, 2024).

Although numerous studies have been done on algal biosorption and response surface methodology-based heavy metal removal, comprehensive mechanistic investigations incorporating process optimization with adsorption equilibrium, kinetic, thermodynamic, and surface characterization for zinc biosorption by marine brown alga *Ericaria selaginoides* remain limited (Arifin et al., 2026; Sekaran et al., 2026). To bridge this gap and provide an environmentally appropriate substitute to traditional techniques, the current study combines Box-Behnken optimization with isotherm, kinetic, thermodynamic, scanning electron microscopy, energy-dispersive X-ray spectroscopy, and Fourier transform infrared (FTIR) analyses to provide comprehensive insights into zinc biosorption by *E. selaginoides*. To the best of our knowledge, this is the first investigation to combine these methodologies for this brown macroalga, yielding new information about both optimization and the underlying biosorption mechanism.

## Materials and Methods

### Chemicals and biosorbent

Zinc sulfate (ZnSO_4_) was used as the Zn(II) ion source in all experiments in this study. The stock solution of zinc sulfate was prepared by dissolving 2.47 g in 1 L of distilled water. The algal biomass of brown alga *Ericaria selaginoides* (Linnaeus) Molinari and Guiry was harvested from Red Sea coast of Jeddah, Saudi Arabia, in autumn 2021. This study used algae samples that were readily available and typically occur on these beaches year-round. Algal samples were thoroughly washed with deionized water to remove contaminants, and dried in an oven for 24 h at 60 °C. The dried biomass was then ground to a particles ranging in size from 0.4 to 1.0 mm and stored at room temperature.

### Experimental design

#### Single factor experiments

This study investigated the effects of initial Zn(II) concentration, contact time, and temperature on the biosorption efficiency and capacity of *Ericaria selaginoides*. The influence of initial Zn(II) concentration was examined by preparing solutions with concentrations of 10, 30, 50, 70, and 90 mg/L, achieved by diluting a stock zinc sulfate solution. A fixed amount of biosorbent was added to each solution and agitated under controlled conditions. The effect of contact time was evaluated at a constant initial Zn(II) concentration and biosorbent dose, with samples withdrawn at time intervals of 0, 5, 10, 15, 20, 30, 50, 70, 100, and 120 min under stirring to measure residual zinc concentrations over time. Additionally, the impact of temperature was studied at 15 °C, 25 °C, and 35 °C, with all other parameters held constant, and residual zinc concentrations measured after a fixed contact period to determine how temperature affects biosorption process (Alharbi et al., 2022; Tao et al., 2024).

#### Optimization experiment

To optimize the biosorption process, considering the interaction of multiple factors, the Box-Behnken design (BBD) using response surface methodology (RSM) was employed. Factors and their levels included algae dose (0.1, 0.3, and 0.5 g/100 mL), initial Zn(II) concentration (30, 50, and 70 mg/L), and pH (3, 5, and 7). The values of the three levels coded as −1, 0, 1 (low, medium, and high, respectively). In the current study, the total number of BBD-based trials was 15. Experiments were conducted at a fixed contact time of 50 min and a temperature of 25 °C, under stirring conditions identical to those used in the single factor experiments, with each trial performed three times, and the data were expressed as mean ± standard deviation.

The relationship between zinc removal efficiency (dependent parameter) and the independent variables was determined using a quadratic multiple regression equation, which is represented as:

(1) \begin{eqnarray*}Y={\mathrm{\beta }}_{0}+\sum _{i=1}^{k}{\mathrm{\beta }}_{i}{\mathrm{X}}_{i}+\sum _{i=1}^{k}{\mathrm{\beta }}_{ii}{\mathrm{X}}_{i}^{2}+\sum _{i< j}^{k}{\mathrm{\beta }}_{ij}{\mathrm{X}}_{i}{\mathrm{X}}_{j}+\end{eqnarray*}

where Y is the response (% of zinc removal), and β_0_, β_i_, β_i_ and β_ij_ are the regression coefficients of variables for intercept, linear, quadratic, and interaction factors, respectively. The independent variables are X_i_ and X_j_, k is the number of independent variables, while the residual term is *ɛ*.

### Biosorption procedure

For each trial, a specified amount of dried macroalga was added to 100 mL of the prepared zinc solution. Before biosorption, the pH was adjusted to 5. The mixture was stirred at 160 rpm and maintained at 25 °C. After the designated contact time, the biomass was rapidly separated from the solution by filtration through Whatman No. 1 filter paper. Each experiment was repeated three times, and the residual Zn(II) concentration in the supernatant was measured (Shobham et al., 2025).

### Analytical methods

The Zn(II) concentration in the supernatant was determined by atomic absorption spectroscopy (AAS) (Buck Scientific 210VGP, Inc., East Norwalk, CT, USA) employing flame and graphite furnace atomization techniques at an analytical wavelength of 213.9 nm. The instrument detection limit for zinc under the applied conditions was 0.01 µg L^−1^. Quality control was maintained by using distilled water as a blank and by calibrating the instrument within the specified zinc concentration range of 0.1–1.5 µg L^−1^ (Jamiu et al., 2025). All measurements were conducted in triplicate, and the data are described as mean ± standard deviation.

The zinc biosorption capacity (q_e_) and efficiency (%) were determined using Eqs. (2) and (3):

(2) \begin{eqnarray*}{\mathrm{q}}_{\mathrm{e}}(\mathrm{mg}/\mathrm{g})= \frac{\mathrm{V}({\mathrm{C}}_{0}-{\mathrm{C}}_{\mathrm{f}})}{\mathrm{W}} \end{eqnarray*}

(3) \begin{eqnarray*}\text{Biosorption efficiency}(\%)= \frac{{\mathrm{C}}_{0}-{\mathrm{C}}_{\mathrm{f}}}{{\mathrm{C}}_{0}} \times 100\end{eqnarray*}

where C_0_ (mg/L) and C_f_ (mg/L) represent the initial and final Zn(II) concentration at any time, V represents the volume of solution per mL, and W (g/L) is the amount of dried biomass.

### Models study

#### Isotherm models

In adsorption isotherms experiments, 100 mL solutions were prepared in which zinc concentrations varied from 10 mg/L to 90 mg/L. Other conditions were kept constant such as algae dose 0.1 g/100 mL, pH 5, and contact time 50 min at 25 °C. Each experiment was carried out in triplicate. The residual Zn(II) concentration in the supernatant was measured as mentioned in the previous section.

##### Langmuir model.

The Langmuir biosorption model explains how molecules adhere to a homogeneous surface with a finite number of identical sites (Ragadhita & Nandiyanto, 2022). Its main parameters include the maximum adsorption capacity (*q*_*max*_), the affinity constant (*b*), and the separation factor (*R*_*L*_), which indicates whether adsorption is favorable. The model’s equation relates the amount adsorbed at equilibrium to the concentration in solution, helping to understand sorption behaviour and capacity (Eqs. (4) and (5)).

(4) \begin{eqnarray*} \frac{{\mathrm{C}}_{\mathrm{eq}}}{{\mathrm{q}}_{\mathrm{e}}} = \frac{1}{{\mathrm{q}}_{\mathrm{max}}\mathrm{b}} + \frac{{\mathrm{C}}_{\mathrm{eq}}}{{\mathrm{q}}_{\mathrm{max}}} \end{eqnarray*}

(5) \begin{eqnarray*}{R}_{L}= \frac{1}{1+b{C}_{0}} \end{eqnarray*}

where *C*_0_ is the initial Zn(II) concentration, *C*_*eq*_ is the final Zn(II) concentration at any time, and *q*_*e*_ is the amount of zinc adsorbed at equilibrium.

##### Jovanovic model.

The Jovanovic isotherm model approximates localized monolayer adsorption by assuming the lack of lateral interactions, making it comparable to the Langmuir model. This model is represented as follows:

(6) \begin{eqnarray*}ln{q}_{e}=ln{q}_{max}-{K}_{J}{C}_{eq}\end{eqnarray*}

where *K*_*J*_ is the Jovanovic constant.

##### Freundlich model.

The Freundlich biosorption model describes adsorption on a heterogeneous surface with sites of varying affinities. It is an empirical model widely used to describe adsorption isotherms that do not follow the Langmuir model (Vigdorowitsch et al., 2021). Freundlich model in linear form is as follows:

(7) \begin{eqnarray*}\mathit{ln}{q}_{e}=ln{K}_{f}+ \frac{1}{n} ln{C}_{eq}\end{eqnarray*}

where *K*_*f*_ (mg/g) is the Freundlich constant which indicates adsorption capacity, and *n* is the heterogeneity factor.

##### Dubinin-Radushkevich (D-R) model.

The D-R sorption model is used to describe adsorption processes, especially to distinguish between physical and chemical adsorption (Chu et al., 2024). It assumes a Gaussian energy distribution on a heterogeneous surface. D-R equation is expressed as:

(8) \begin{eqnarray*}ln{q}_{e}=ln{q}_{0}-\beta {}^{2}\end{eqnarray*}

where *q*_*e*_ is the amount of zinc adsorbed at equilibrium, *q*_0_ is the theoretical saturation capacity, and β is the D-R isotherm constant related to mean adsorption energy. *ɛ* (Polanyi potential) and *E* (the average free energy of biosorption) are calculated as:

(9) \begin{eqnarray*}=RT\mathit{ln}(1+ \frac{1}{{C}_{eq}} )\end{eqnarray*}

(10) \begin{eqnarray*}E=(2\beta )^{-1/2}\end{eqnarray*}

where *C*_*eq*_ is the equilibrium concentration, *R* is the universal gas constant and *T* is the absolute temperature.

##### Temkin model.

The Temkin adsorption model describes adsorption in which the heat of adsorption decreases linearly with increasing coverage due to adsorbate-adsorbent interactions (Zhou, Maimaitiniyazi & Wang, 2022). It assumes a uniform distribution of binding energy until a certain maximum binding energy is reached. Temkin equation in a linear form is represented as follows:

(11) \begin{eqnarray*}{q}_{e}=B~lnA+B~ln{C}_{eq}\end{eqnarray*}

(12) \begin{eqnarray*}B= \frac{RT}{b} \end{eqnarray*}

where *B* relates to the heat of adsorption, *b* is the constant Temkin isotherm model, *A* is the equilibrium binding constant (L/mg), *R* is the universal gas constant and *T* is the absolute temperature.

### Kinetic models

Biosorption kinetic investigations were conducted in 250 mL flasks containing 100 mL of distilled water, under the established conditions of pH 5, zinc content of 50 mg/L, and algal dosage of 0.1 g/100 mL at 25 °C. Each treatment was repeated three times, and samples were collected and analyzed at intervals of 0, 5, 10, 15, 20, 30, 50, 70, 100, and 120 min to assess zinc concentrations over time.

#### Pseudo-first-order model

The model assumes the occupancy rate of biosorption sites is directly proportional to the number of unoccupied sites (Subash et al., 2023). The pseudo-first order model is expressed in its linear form as follows:

(13) \begin{eqnarray*}\log \nolimits \left( {q}_{e}-{q}_{t} \right) =\log \nolimits ~{q}_{e}- \frac{{k}_{1}}{2.303} t\end{eqnarray*}

where *q*_*e*_ and *q*_*t*_ are the amounts of zinc adsorbed at equilibrium and at time *t* (mg/g), respectively, *t* is the contact time (min), and *k*_1_ is the rate constant of pseudo-first-order biosorption (1/min).

#### Pseudo-second-order model

This model assumes that chemisorption is the rate-limiting step of the reaction, and the linear form of this model is expressed as follows:

(14) \begin{eqnarray*} \frac{t}{{q}_{t}} = \frac{1}{{k}_{2}{q}_{e}^{2}} + \frac{1}{{q}_{e}} t\end{eqnarray*}

where *q*_*e*_ and *q*_*t*_ are the amounts of zinc adsorbed at equilibrium and at time *t* (mg/g), respectively, and *k*_2_ is the rate constant of pseudo-second order model (g/mg min) (Wang, Zhao & Hou, 2024; Fouad & Mohamed, 2024).

#### Elovich model

This model is often used for chemical adsorption on heterogeneous surfaces (Eq. (15)):

(15) \begin{eqnarray*}{q}_{e}= \frac{1}{\mathrm{\beta }} \ln \nolimits ~ \left( \alpha \mathrm{\beta } \right) + \frac{1}{\mathrm{\beta }} \ln \nolimits ~t\end{eqnarray*}

where *q*_*e*_ is the amount of zinc adsorbed at equilibrium (mg/g), *α* (mg/g.min) is the initial adsorption rate, β (g/mg) is Elovich constant related to the extent of surface coverage and activation energy for chemisorption, and *t* is the contact time (min) (Inyang, Onwawoma & Bae, 2016).

#### Intra-particle diffusion model

To evaluate the diffusion mechanism, an intra-particle diffusion model was applied (Eq. (16)):

(16) \begin{eqnarray*}{q}_{t}={k}_{i}{t}^{1/2}+{C}_{i}\end{eqnarray*}

where *q*_*t*_ (mg/g) is the adsorption capacity at time *t*, *k*_*i*_ (mg g^−1^ min^−1/2^) is the intra-particle diffusion rate constant, *t*^1/2^ is the square root of contact time, and *C*_*i*_ (mg/g) reflects the boundary layer thickness (Nguyen et al., 2015).

### Thermodynamic analysis

The thermodynamic studies were evaluated at different temperatures (15 °C or 288 K, 25 °C or 298 K, and 35 °C or 308 K) and fixed conditions of algae dose of 0.1 g/100 mL, pH 5, zinc concentration of 50 mg/L, and contact time of 50 min. The zinc concentration in the solution was determined as described previously.

To elucidate the nature and feasibility of the biosorption process, thermodynamic parameters such as distribution coefficient (K_d_), Gibbs free energy change (ΔG^∘^), enthalpy change (ΔH^∘^), and entropy change (ΔS^∘^) were evaluated based on experimental data (Hernández-Rizo, Larios-Durán & Bárcena-Soto, 2023). Thermodynamic parameters were calculated from equilibrium adsorption data at various temperatures. The following equations were employed:

(17) \begin{eqnarray*}{k}_{d}= \frac{{q}_{e}}{{C}_{e}} \end{eqnarray*}

where *q*_*e*_ is the amount of zinc adsorbed per unit weight of biosorbent at equilibrium (mg/g), and *C*_*e*_ is the equilibrium concentration of zinc in solution (mg/L).

The standard Gibbs free energy change was calculated from the following equation:

(18) \begin{eqnarray*}\Delta {G}^{0}=-RT~ln{k}_{d}.\end{eqnarray*}

The relationship among the standard Gibbs free energy, enthalpy, and entropy changes is given by:

(19) \begin{eqnarray*}\Delta {G}^{0}=\Delta {H}^{0}-T\Delta {S}^{0}\end{eqnarray*}

where *R* is the universal gas constant (8.314 J/mol.K), and *T* is the absolute temperature in Kelvin.

The values of ΔH^∘^ and ΔS^∘^ were determined from the linearized van’t Hoff equation as follows:

(20) \begin{eqnarray*}\ln \nolimits {k}_{d}= \frac{\Delta {S}^{0}}{R} - \frac{\Delta {H}^{0}}{RT} .\end{eqnarray*}

### Characterization of biosorbent

The surface morphology of *E. selaginoides* was examined using scanning electron microscopy (SEM; JEOL, JSM-5400 LV). The elemental composition was analyzed using energy-dispersive X-ray analysis (EDXA; JEOL, JSM-IT 200). The functional groups involved in zinc biosorption were identified using Fourier transform infrared spectroscopy (FT-IR; Bio-Rad, FTS 3000 MX) (Thamarai et al., 2025).

### Data analysis

All models were fitted to the experimental data using linear regression analysis. The correlation coefficient (R^2^) was used to evaluate the goodness of fit. The model with the highest R^2^ value was considered the most appropriate to describe the biosorption. Design-Expert software (Stat-Ease, version 7.0, Inc., Minneapolis, MN, USA) was used to develop statistical analysis, regression equations, and response surface plots of RSM to determine the best zinc removal conditions.

**Figure 1:**
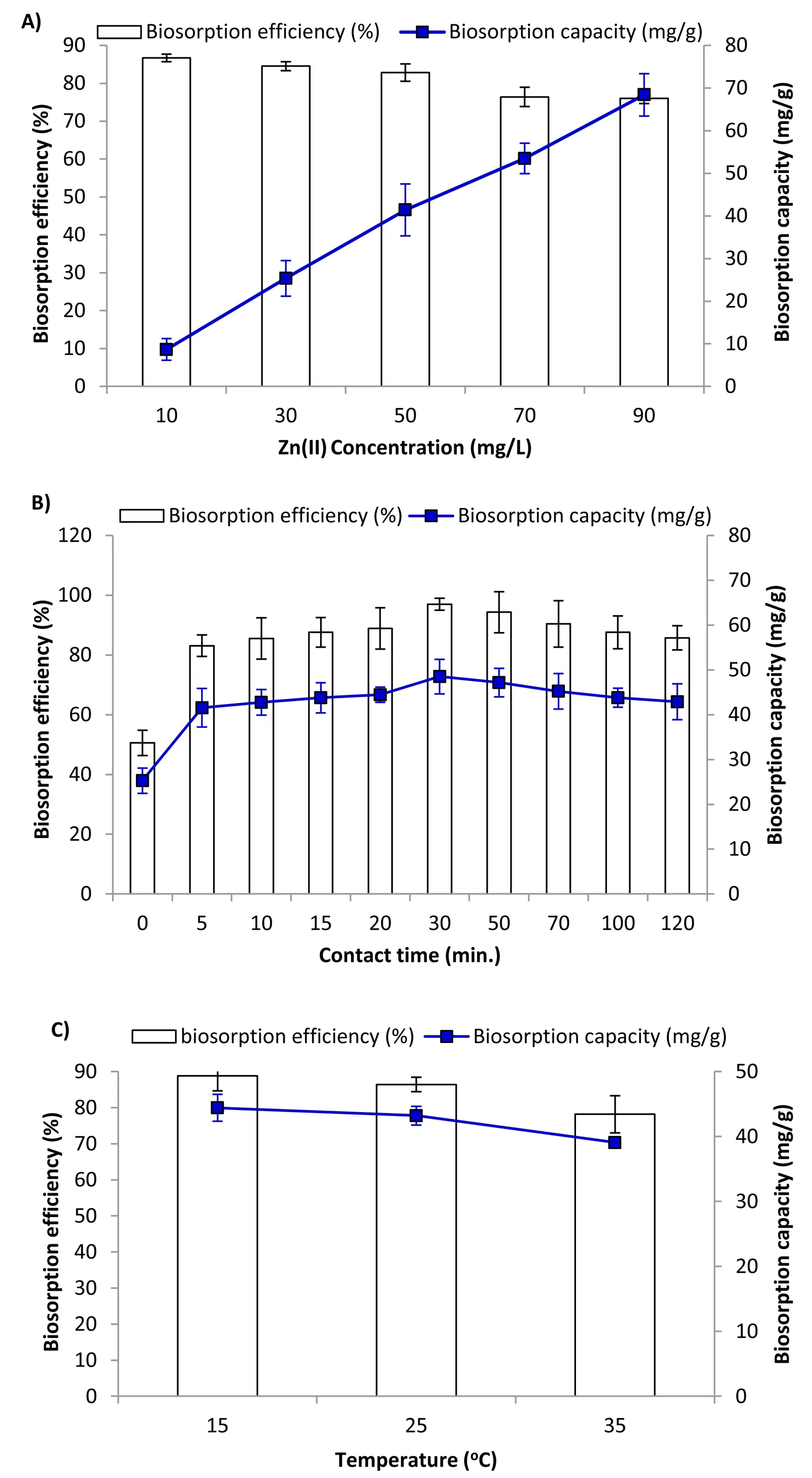
Effect of different independent parameters on removal of Zn(II) by *E. selaginoides* (A) zinc concentration, (B) contact time, and (C) temperature.

## Results and discussion

### Single factor experiments

Figure 1 shows the effects of various parameters on the biosorption process by *E. selaginoides*. Figure 1A shows that as the zinc ion concentration increases from 10 to 90 mg/L, the amount of Zn(II) adsorbed onto *E. selaginoides* increases steadily from 8.67 mg/g to 68.41 mg/g, whereas the percentage of Zn(II) removal decreases at higher concentrations. The observed increase in biosorption capacity is consistent with previous studies, which showed that higher metal ion availability enhances biosorption to saturation levels (Parmar & Patel, 2025). According to Wang et al. (2015), a high initial metal concentration lowers the mass transfer resistance and speeds up the driving force. On the other hand, the decrease in biosorption efficiency at elevated concentrations can be elucidated by the fact that the algal biomass contained a small number of active binding sites that were saturated at a certain concentration (Hassan et al., 2025).

Figure 1B displays that increasing the contact time from 0 to 30 min causes a rapid initial increase in biosorption capacity, which then gradually stabilizes, indicating that equilibrium is reached around 30 min; similarly, the removal efficiency initially increases and then stabilizes. The rapid initial uptake followed by stabilization, suggest a typical pattern of biosorption kinetics associated with film and pore diffusion mechanisms (Ho & McKay, 1999). This is due to the fact that the surface of algal biomass contains many active sites responsible for zinc biosorption during the early stages of contact time. These available binding sites facilitate the interaction and adsorption of Zn(II) ions on the algal surface, resulting in rapid removal kinetics (Olawale et al., 2022). The stabilization after about 30 min indicates that further uptake was limited by the reduced availability of active binding sites and increasing resistance to mass transfer (Al-Mijalli, 2022). In a related investigation, Mohammed, Shartooh & Trigui (2025) found that 30 min was the optimum contact time for *Phragmites australis* biomass to biosorb heavy metals.

Figure 1C indicates that increasing the temperature from 15 °C to 35 °C causes a decrease in both the biosorption capacity and removal efficiency. This can be attributed to the exothermic nature of the biosorption process. Increasing temperature may reduce the thermodynamic favorability of Zn(II) uptake and weaken the affinity between zinc ions and the available binding sites on the algal biomass, resulting in lower Zn(II) retention at higher temperatures (Ngah & Hanafiah, 2008).

Understanding the influence of these parameters is crucial for designing efficient biosorption systems for heavy metal remediation, and further studies could investigate the mechanistic aspects and performance of *E. selaginoides* under varying environmental conditions.

### Box–Behnken analysis

A total of fifteen trials were conducted using three experimental variables: biosorbent dose (g/mL), biosorbate concentration (mg/L), and pH, to assess their individual and interactive effects on the percentage of zinc ion removal. Table S1 lists the experimental and coded values of the independent parameters, and the actual and expected removal percentages. Figure 2A also shows the difference between the actual and predicted values obtained from the RSM model. The graphic shows that the accuracy of the provided semi-empirical model may be shown by considering the appropriate concentration of the actual and predicted biosorption efficiency values along the diagonal line. Furthermore, the accuracy of the proposed model is expected by the linear distribution of values and the lack of outliers in the normal plot of the model residues (Fig. 2B). Figure 2C indicates that residuals were randomly distributed around the central line, demonstrating high agreement between experimental and expected values of Zn(II) biosorption. Furthermore, the residuals were within the range of ±3, indicating that no response transformation was required for the design (Hassan et al., 2025).

The relationship between the independent variables in coded units and the response (% zinc removal) were examined using a second-degree polynomial equation (Eq. (21)).

(21) \begin{eqnarray*}R\%=83.63+1.27\mathrm{A}-3.28\mathrm{B}+2.54\mathrm{C}+3.77\mathrm{AB}-1.75\mathrm{AC}+1.21\mathrm{BC}-4.92{\mathrm{A}}^{2}-\nonumber\\\displaystyle 1.82{\mathrm{B}}^{2}+2.9{\mathrm{C}}^{2}\end{eqnarray*}

where A, B, and C represent algal dose, initial zinc concentration, and pH, respectively.

The analysis of variance (ANOVA) data (Table 1) reveal that the overall model has a high sum of square (*SS* = 364.99), an *F* value of 11.38, and a *p* value of 0.0077, confirming its significance in explaining the variability in zinc biosorption. Among the variables, biosorbate concentration (B) has a high *SS* value (85.81), a very low *p* value (0.0044), and a large negative effect (*CE* = −3.28), indicating that increasing the initial zinc concentration reduced the percentage removal. In contrast, pH (C) shows a significant positive effect (CE = 2.54) with a *p*-value of 0.0126, indicating that increasing pH within the studied range favored zinc removal. The interaction between biosorbent dose and biosorbate concentration (AB) is also significant (*p* = 0.0104), indicating that the influence of one variable depended on the level of the other. Furthermore, the quadratic terms for the algae dose (A^2^) and pH (C^2^) are significant (*p* < 0.05), suggesting a curvature effect on the response surface. These results are consistent with previous research highlighting the importance of pH and biosorbent dose in heavy metal biosorption (Dekhil et al., 2011).

**Figure 2:**
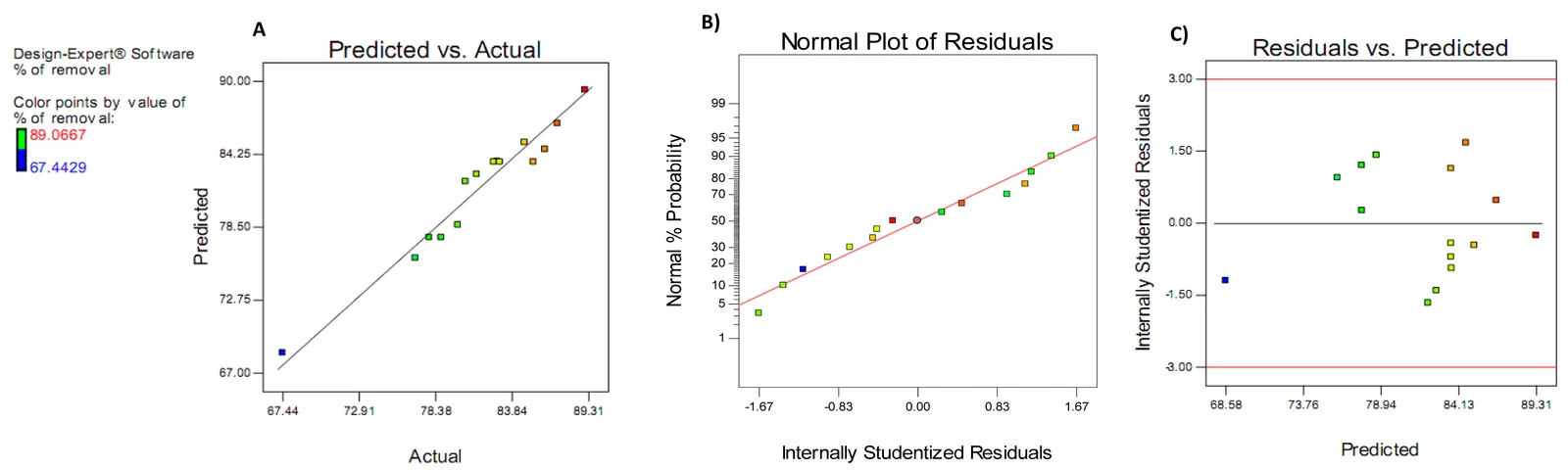
(A) The actual *versus* predicted plot, (B) the normality plot, and (C) Internally studentized residual *versus* predicted response plot of Zn(II) ion biosorption by *E. selaginoides*.

**Table 1:** ANOVA, regression coefficient estimate, significance test for Zn(II) removal onto *E. selaginoides*.

Source	Coefficient estimate	Sum of squares	Degree of freedom	Mean square	*F*-value	*p*-value Prob > F
Model	–	364.99	9	40.55	11.38	0.0077^*^
A-Biosorbent dose	1.27	12.93	1	12.93	3.63	0.1152
B-Biosorbate conc.	−3.28	85.81	1	85.81	24.08	0.0044^*^
C- pH	2.54	51.52	1	51.52	14.46	0.0126^*^
AB	3.77	56.82	1	56.82	15.95	0.0104^*^
AC	−1.75	12.25	1	12.25	3.44	0.1229
BC	1.21	5.87	1	5.87	1.65	0.2554
A^2^	−4.92	89.42	1	89.42	25.1	0.0041^*^
B^2^	−1.82	12.25	1	12.25	3.44	0.1229
C^2^	2.9	31.01	1	31.01	8.7	0.0319^*^
Residual	–	17.82	5	3.56		
Lack of Fit	–	13.14	3	4.38	1.87	0.3664^**^
Pure Error	–	4.67	2	2.34	–	–
Cor. Total	–	382.81	14	–	–	–
R^2^	0.953					
R^2^-adjusted	0.870					
R^2^-predicted	0.723					
% Coefficient of variation.	2.314					
Adequate precision	13.45					

**Notes.**

* Significant at *p* < 0.05.

** Not significant at *p* > 0.05.

The lack of fit has a *p*-value of 0.366, indicating that the model fits the data well without significant deviation. The adjusted *R*^2^ (0.870) and predicted *R*^2^ (0.723) further show acceptable agreement between the model and the experimental response, although the lower predicted *R*^2^ indicates some loss of predictive performance when going beyond the fitted results (Hassan et al., 2025).

The residuals account for the unexplained variability (SS = 17.82), with the residual degree of freedom being 5 and the pure error (SS = 4.67, *df* = 2) reflects experimental error. In addition, the ratio of the residuals to the pure error shows the model’s precision. The low coefficient of variation value (% C.V. = 2.314) indicates good experimental precision, while the Adequate Precision value of 13.45, which is substantially higher than the recommended value of 4, shows an adequate signal-to-noise ratio for navigating the design space (Thakur & Gupte, 2015).

**Figure 3:**
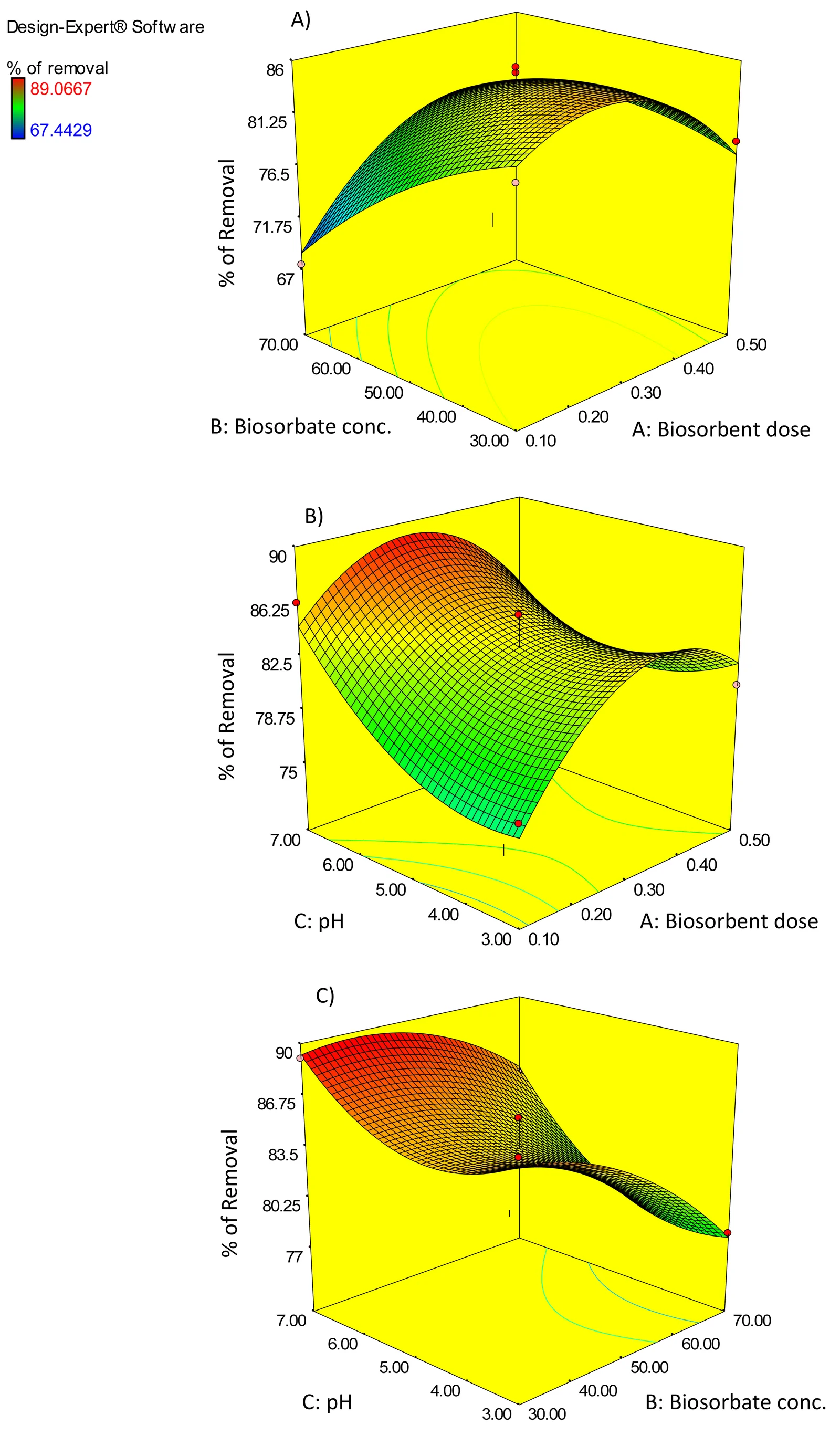
(A–C) Response surface plots displaying the interaction impact between different parameters on Zn(II) biosorption onto *E. selaginoides*.

### Three-dimensional response surface

The 3-D response surfaces in Fig. 3 show the significant combinations among the independent variables and provide a graphical explanation of the quadratic model. Figure 3A analyses the combined effect of biosorbent dose and biosorbate concentration. The percentage of zinc removal increased significantly with increasing biosorbent dose, particularly at lower Zn(II) concentrations, and reached an optimal removal of about 82.95% within specific levels of these variables (biosorbent dose ≈ 0.30–0.35 g/100 mL and zinc concentration ≈ 35 mg/L). The positive impact of biosorbent dose can be ascribed to the increased number of active sites on the surface of the algal biomass and greater biosorbent–solution contact area (Chakraborty et al., 2022). However, beyond a certain dose the curve begins to reach a plateau, indicating that either the system is approaching the saturation stage or the excess biosorbent no longer makes a noticeable difference. In contrast, increasing the zinc concentration led to a decrease in removal efficiency due to the gradual occupation of available active sites (Fouad, El-Aswad & Aly, 2025).

Figure 3B shows a graph describing the interaction of algal dose and pH on the percentage of zinc ion removal. Zinc removal increased with increasing pH, particularly at higher algal doses, reaching nearly 86.88% at pH 7. Adjusting pH can alter the biosorbent’s surface charge and the speciation of metal ions, thereby affecting adsorption capacity (Mohammed, Shartooh & Trigui, 2025). Algae can exchange ions with positively charged metals in aqueous solution because they contain more free carboxylate groups (-COO^−^), which become less accessible at lower pH levels as they protonate and become -COOH. This reduces adsorption by reducing the ability of zinc ions to form bonds. The observed increase in Zn(II) biosorption by the algal biomass with rising pH values can be attributed to changes in the algal surface, which influence the interaction of metal complex ions with cells. These interactions occur mostly through complex formation, electrostatic forces, electron-sharing mechanisms, or alterations in membrane transport (Kumari et al., 2023). Furthermore, at higher pH values, competition with hydrogen ions decreases, allowing for more efficient adsorption.

Figure 3C demonstrates the interaction between biosorbate concentration and pH. The data show that the percentage of zinc ion removal increases significantly with increasing pH at low zinc concentrations, reaching approximately 89%. At higher concentrations of zinc ions, the positive impact of pH was less pronounced, reflecting the limited number of available active sites compared to the amount of zinc(II) ions. This interaction is consistent with the significant impacts obtained by the quadratic model and demonstrates that the effect of pH cannot be considered independently of the zinc concentration.

Generally, the response surface methodology confirms that zinc removal is governed by the mutual impacts of algal dose, pH, and zinc concentration rather than by any single parameter. Therefore, the response surface analysis provided the basis for numerical optimization using the desirability function.

### Optimization using desirability functions

In the Zn removal process, achieving maximum removal was the primary goal of the experimental analyses. The desirability functions are crucial for calculating the optimal target for each parameter and response. The predicted optimal conditions for maximum removal efficiency by algal biomass were obtained at a biosorbent dose of 0.28 g/100 ml, a biosorbate concentration of 33.62 mg/l, and pH 6.97 at a desirability value of 1.00. To validate the numerical optimization, an independent trial was performed under these conditions. The experimentally obtained zinc removal percentage was 87.72 ± 7.3% (Table 2), which is consistent with the predicted value of 89.79%, corresponding to a percentage error of 2.31%. The close agreement between experimental and predicted values demonstrates the validity of RSM in optimizing biosorption conditions (Fertu, Bulgariu & Gavrilescu, 2022). The experimental validation further shows that the optimum circumstances achieved from the desirability function were experimentally feasible.

**Table 2:** Optimum variables of removal of Zn(II) by *E. selaginoides*.

Variables			% removal of Zinc ions	
A: Biosorbent dose (g/100 mL)	B: Biosorbate conc. (mg/L)	C: pH	Actual	Predicted
0.28	33.62	6.97	87.72 ± 7.3	89.79

### Biosorption isotherm and kinetic models

Table 3 and Fig. S1 provide the isotherm parameters obtained from the Langmuir, Jovanovic, Freundlich, Dubinin–Radushkevich, and Temkin models, while Fig. S2 shows the kinetic fits obtained using the pseudo-first order (PFO), pseudo-second order (PSO), Elovich, and intra-particle diffusion models. These models were used to elucidate the efficiency, mechanism, and capacity of the biosorption process (El-Aswad et al., 2024).

**Table 3:** Isotherm and kinetic parameters for biosorption of Zn(II) onto *E. selaginoides* biomass.

Models	Parameters	Values
Isotherms		
Langmuir	*q*_*max*_ (mg/g)	114.94
	*b* (L/mg)	0.06
	*R*_*L*_	0.135–0.385
	*R* ^2^	0.962
Jovanovic	*q*_*max*_ (mg/g)	12.83
	*K*_*J*_ (L/mg)	0.087
	*R* ^2^	0.791
Freundlich	*K*_*f*_ (mg/g)	7.629
	*n*	1.38
	*R* ^2^	0.985
Dubinin and Radushkevich	*q*_*o*_ (mg/g)	48.52
	*β*×10^−7^(mol^2^/J^2^)	9.0
	*E* (kJ/mol)	0.746
	*R* ^2^	0.873
Temkin	*B*	20.505
	*A* (L/mg)	1.064
	*b* (J/mol)	120.83
	*R* ^2^	0.957
Kinetics		
Experimental data	*q* (exp.) (mg/g)	48.50
Pseudo-first order	*q* (cal.) (mg/g)	15.36
	*K*_1_ (min^−1^)	0.078
	*R* ^2^	0.778
Pseudo-second order	*q* (cal.)(mg/g)	44.64
	*K*_2_(g/mg min)	0.102
	*R* ^2^	0.999
Elovich	*α* (g/mg min)	9.806
	*β* (g/mg)	0.156
	*R* ^2^	0.904
Intra-particle diffusion	*K*_*i*1_ (mg g^−1^min^−1/2^)	3.496
	*K*_*i*2_ (mg g^−1^min^−1/2^)	0.336
	*C*_*i*1_ (mg/g)	27.79
	*C*_*i*2_ (mg/g)	41.14
	*R* ^2^_1_	0.802
	*R* ^2^_2_	0.985

The Langmuir isotherm (Table 3) indicates a maximum biosorption capacity (*q*_*max*_) of 114.94 mg/g and a binding affinity constant (*b*) of 0.06 L/mg, with a favourable separation factor (*R*_*L*_) ranging from 0.135 to 0.385 and a high *R^2^* value of 0.962 (Fig. S1A), suggesting a dominant monolayer chemical adsorption process driven by surface complexation and ion exchange mechanisms.

The Jovanovic isotherm model (Fig. S1B) yielded a maximum biosorption capacity of 12.83 mg/g. Furthermore, the comparatively low regression coefficient value (*R*^2^ = 0.791) suggests that there are lateral interactions and no mechanical interactions between the Zn(II) ions and the biomass of *E. selaginoides*. Consequently, the Jovanovic model shows a lower tendency toward saturation than the Langmuir isotherm model (Fawzy et al., 2022).

The Freundlich model (Table 3) also fits well (*R^2^* = 0.985; Fig. S1C), with an adsorption capacity (*K*_*f*_) of 7.629 mg/g and a heterogeneity factor (*n*) of 1.38, indicating favourable biosorption and possible chemisorption and/or physisorption interactions. This good agreement between the Langmuir and Freundlich models suggests that Zn(II) biosorption predominantly occurred as monolayer adsorption on a heterogeneous biosorbent surface with adsorption sites of different binding energies.

The Dubinin and Radushkevich (D-R; Fig. S1D) model reported a biosorption capacity (*q*_0_) of 48.52 mg/g and a low adsorption energy (*E*) of 0.746 kJ/mol (<8 kJ/mol), implying a considerable contribution of weak physical interactions to the zinc biosorption process (Ho & McKay, 1999). However, this model should not be considered sufficient by itself to establish that the entire biosorption process is purely physical.

The Temkin model showed a relatively good fit (*R*^2^ = 0.957; Fig. S1E), with a *B* value of 20.505, further supporting the occurrence of interactions between zinc ions and the biosorbent surface.

The kinetic data provide further evidence for the complexity of the biosorption process. The PSO model gave the best fit (*R^2^* = 0.999; Table 3), with the calculated capacity (44.64 mg/g) closely matching the experimental value (48.50 mg/g) and a rate constant (*K*_2_) of 0.102 g/mg min, indicating rapid kinetics dominated by chemisorption (Fig. S2B). In comparison, the PFO model exhibits a lower *R^2^* (0.778; Fig. S2A), suggesting that it is less suitable for describing the process.

The aforementioned results suggest that the biosorption of zinc onto the *E. selaginoides* biomass should be interpreted as a combined physicochemical process rather than being governed exclusively by either physisorption or chemisorption.

The high correlation coefficient (*R*^2^ = 0.904; Fig. S2C) of the Elovich model suggests that the biosorption process was controlled by both reaction rate and diffusion (Robati et al., 2016). The value of the rate constant (*α*) is similar to that found by Lavado-Meza et al. (2022), which can be ascribed to the negative surface charge of the algal biomass that can facilitate zinc uptake.

Intra-particle diffusion analysis (Fig. S2D) demonstrated that the biosorption process takes place in two stages. The higher *K*_*i*1_ value compared to *K*_*i*2_ (Table 3) implies a faster initial uptake of Zn(II), which is mostly caused by external diffusion and biosorption onto easily available active sites of the algal biomass (Hu et al., 2019). As these active sites become more occupied in the first step, the biosorption rate slows and reaches equilibrium, corresponding to the second step, which is related to pore or internal diffusion within the biosorbent particles (Shen et al., 2018). The second step is identified as the rate-controlling stage during the biosorption process. However, because the intercept of the model does not pass *via* the origin, intra-particle diffusion cannot be regarded as the only rate-controlling process (Nessiani et al., 2025). As a result, Zn(II) is biosorption controlled both internally and externally during the algal adsorption process.

### Thermodynamic studies

Thermodynamic parameters derived from temperature-dependent studies further elucidate the feasibility and nature of the biosorption process (Mostafa et al., 2025). Table 4 presents the thermodynamic parameters associated with zinc ions biosorption by *E. selaginoides* at different temperatures (288, 298, and 308K). Examination at different temperatures helps to understand how thermal conditions affect biosorption and the thermodynamic nature of this process. ΔG^∘^ (Gibbs free energy change, kJ mol^−1^) indicates the spontaneity of the biosorption process at each temperature (Pan et al., 2018). Negative values (observed at 288 and 298 K) suggest that zinc biosorption occurs spontaneously under these temperatures. However, the less negative ΔG^∘^ value at 308 K indicates that the thermodynamic driving force for biosorption gradually decreases with increasing temperature, suggesting that although the process remains thermodynamically feasible, its spontaneity becomes less favorable at higher temperatures.

**Table 4:** Thermodynamic parameters for biosorption of Zn(II) onto *E. selaginoides* biomass.

Temperature (K)	ΔG^o^ (kJ mol^−1^)	ΔS^o^ (kJ mol^−1^ K^−^^1^ )	ΔH^o^ (kJ mol^−1^)	R^2^
288	−4.954			
298	−4.577	−0.084	−29.200	0.929
308	−3.259			

ΔS^∘^ (standard entropy change, kJ mol^−1^ K^−1^) reflects the degree of disorder or randomness associated with the biosorption process (Kojima & Yamaguchi, 2021). The negative ΔS^∘^ value (−0.084 kJ mol^−1^ K^−1^) indicates a decrease in randomness, implying that biosorption leads to a more ordered system, likely due to the binding of zinc ions to specific sites on the *E. selaginoides* surface. The negative value of enthalpy change (ΔH^∘^ = −29.200 kJ mol^−1^) indicates that the process is exothermic, indicating that heat is released during Zn^2+^ uptake (Nie et al., 2022). Consequently, increasing the temperature reduces the thermodynamic driving force for adsorption, in accordance with Le Chatelier’s principle, resulting in less negative ΔG^∘^ values and a gradual decline in the spontaneity of the biosorption process at higher temperatures. This thermodynamic behavior is characteristic of exothermic adsorption systems, in which elevated temperatures become progressively less favorable for adsorbate binding. Furthermore, the relatively high *R^2^* value (0.929 at 288 K) indicates a satisfactory fit of the thermodynamic model, supporting the reliability of the calculated thermodynamic parameters (Gao, 2024).

### Structural characteristics of *E. selaginoides*

Figure 4 shows the FTIR spectra representing the infrared absorption characteristics of *E. selaginoides* before (Fig. 4A) and after (Fig. 4B) Zn(II) ion removal. FTIR spectra of the biomass show a broad peak at 3,416 cm^−1^, which shifted to 3,422 cm^−1^ upon loading with Zn(II) ion. The involvement of hydrogen bonds in the zinc ions biosorption by *E. selaginoides* biomass is confirmed by this absorption band, which is typically assigned to the stretching vibrations of N–H and O–H groups in amines and alcohols, respectively (Ciobanu, Lucaci & Bulgariu, 2024). Peaks corresponding to the C–H stretching vibrations of phospholipids and lipid fractions occurred on the algal biomass at 2,924 cm^−1^ (Keshtkar et al., 2019). Following Zn^2+^ biosorption, the peak of 1,624 cm^−1^ shifted to 1,635 cm^−1^. This can be attributed to the carboxylate –COO^−^ stretching vibrations of alginic acid on the cell wall structure of the brown algae, suggesting their involvement in the zinc ions biosorption process due to electrostatic attraction or metal ion complexation, which is consistent with the findings of Kang & So (2016). The deformation vibrations of the –C–OH groups in the polysaccharide are responsible for the presence of the peak at 1,424 cm^−1^ (Khan et al., 2016). Moreover, the peak at 1,257 cm^−1^ demonstrates the presence of a P = O phosphate bond, which also verified in earlier research (Gu, Su & Lan, 2022). An absorption peak corresponding to the stretching vibrations of C–O–C in alcohols and carboxylic compounds was observed at 1,034 cm^−1^. Finally, the bands at 823–824 cm^−1^ demonstrate a C-N bond (Id, Id & Marycz, 2018).

The FTIR analysis results show that *E. selaginoides* possesses a variety of functional groups, including hydroxyl, amine, phosphate and carboxylate groups. Differences between spectra before and after Zn^2+^ biosorption suggest that zinc ion removal affects these functional groups, likely altering their vibrational environment, as reflected in shifts and changes in the FTIR peaks intensity. This analysis also provides insights into the chemical interactions involved in zinc binding and removal by algal biomass. Previous studies suggest that algae exhibit a range of heavy metal uptake mechanisms, including complexation with functional groups such as carboxyl, hydroxyl, and amine (Sumiahadi & Acar, 2018).

The SEM micrographs in Fig. 5 illustrate the surface morphology of *E. selaginoides* before (Fig. 5A) and after (Fig. 5B) Zn(II) ion removal. In Fig. 5A, the surface appears highly structured, smooth, and organized with parallel ridges, suggesting intact tissue and the presence of grooves that may contribute to metal binding (Staszak & Regel-Rosocka, 2024). Following zinc biosrption (Fig. 5B), the surface becomes irregular, rough, and porous, with disrupted and eroded features, indicating significant morphological changes likely caused by zinc interaction. Such morphological modifications could enhance the algae’s metal-binding capacity by increasing surface area and porosity (Ali, Khan & Sajad, 2013). These surface alterations may result from the interaction of the zinc ion with surface functional groups, leading to structural erosion or pore development, which may facilitate metal uptake (Ighalo & Adeniyi, 2020). These results are consistent with structural disruptions observed in other phytoremediation studies (Pandey et al., 2022; Aloufi et al., 2024).

**Figure 4:**
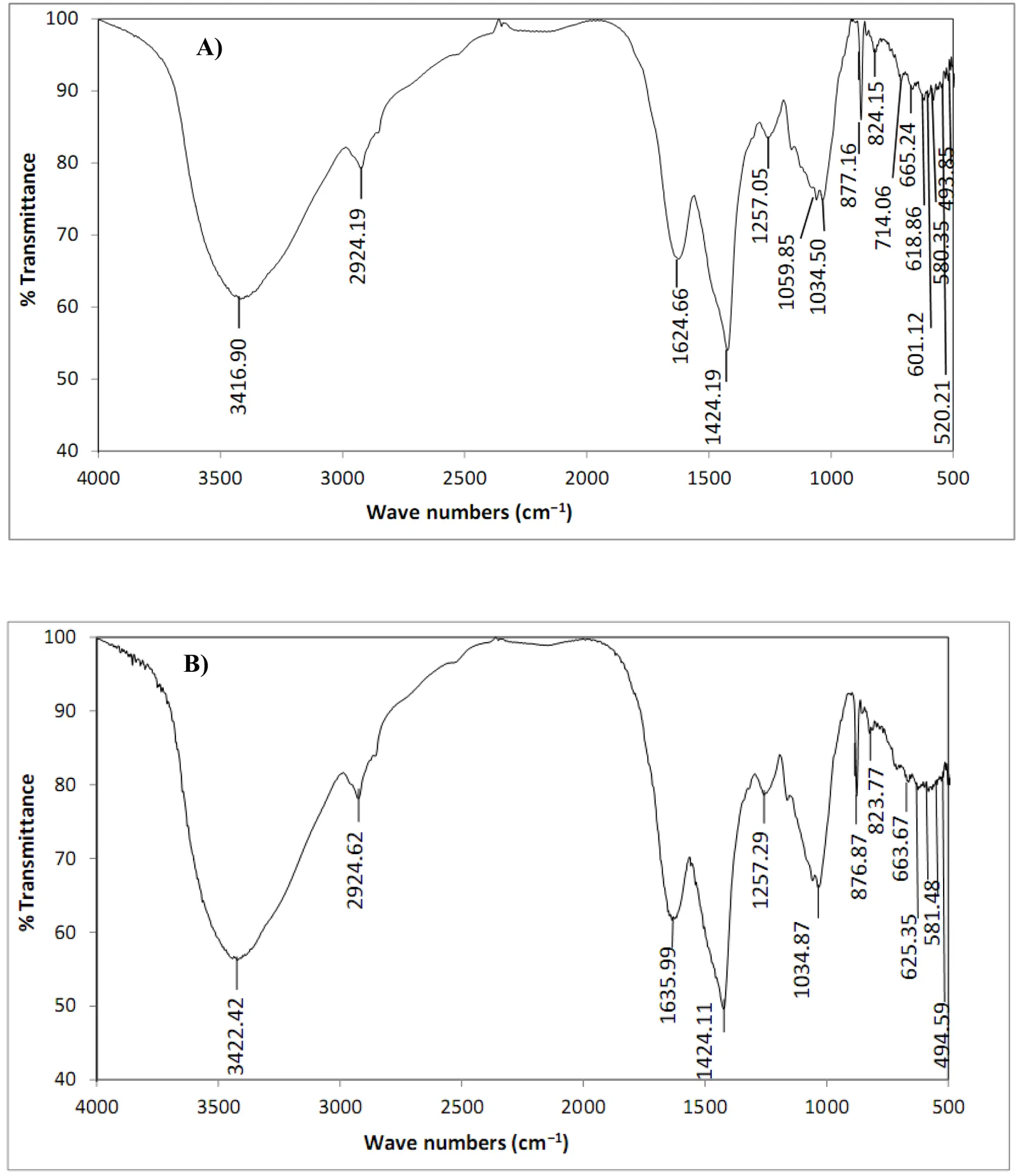
FTIR spectrum of (A) control algal biomass, and (B) zinc adsorbed algal biomass.

**Figure 5:**
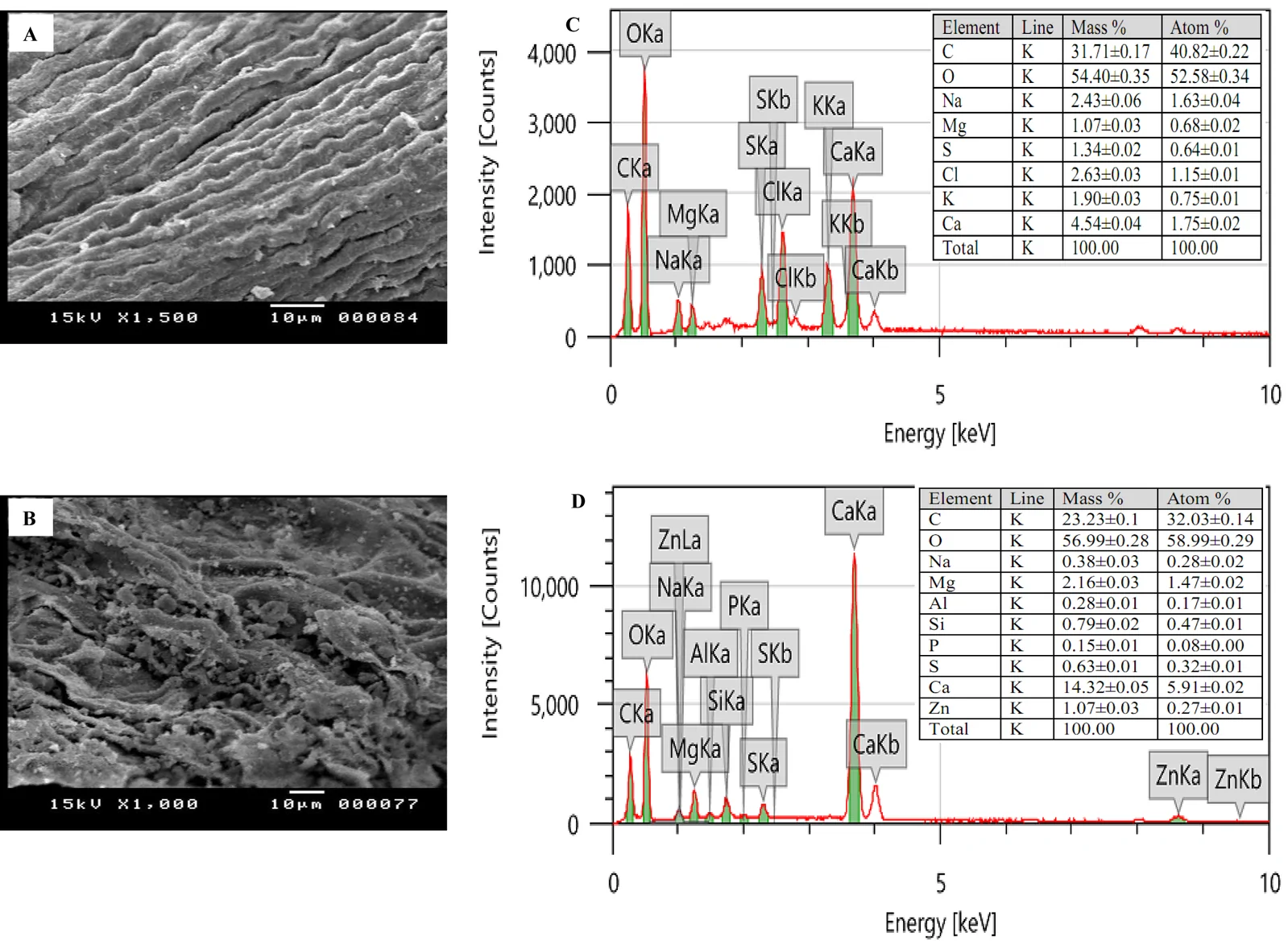
Scanning electron microscopic image of (A) control algal biomass, and (B) zinc adsorbed algal biomass. Energy-dispersive X-ray spectra of (C) control algal biomass, and (D) zinc adsorbed algal biomass.

Figure 5 indicates the results of Energy-Dispersive X-ray analysis (EDXA) analysis of *E. selaginoides* before (Fig. 5C) and after (Fig. 5D) Zn(II) removal, displaying the elemental composition and distribution on the algal surface at different energy levels (keV). The spectrum shown in Fig. 5C exhibits several prominent peaks corresponding to different elements. The most intensive bands are carbon and oxygen, which are common organic components of algal cells, consistent with the composition of plant tissues (Sheue et al., 2020). Additional notable peaks include Mg (magnesium), Na (sodium), Ca (calcium), S (sulphur), Cl (chloride), and K (potassium), reflecting mineral elements naturally present on the algal surface. High atomic percentages for oxygen (40.82%) and carbon (52.58%) suggest a surface rich in organic compounds, while mineral elements contribute to the algae’s structural integrity. After zinc biosorption (Fig. 5D), the spectrum shows replacement and a shift in the surface elemental composition of *E. selaginoides* at some peaks, suggesting that Zn(II) uptake may occur through ion exchange and may also potentially contribute to complex formation with biomass functional groups.

The elemental composition also indicates that zinc ion now appears in measurable atomic percentages, confirming its presence on the algal surface. According to Alharbi et al. (2022), a distinctive zinc peak was observed when *Spirulina platensis* biomass was treated with zinc ions. Zinc-loaded *E. selaginoides* biomass showed a considerable change in the atomic percentages of carbon and oxygen, suggesting that carboxylic and hydroxyl groups on the surface of the algal biomass are crucial for zinc ion adsorption. The changes in peak intensity and the presence of zinc in the spectrum after treatment confirm the effectiveness of zinc removal and highlight the elemental alterations on the surface.

### Proposed zinc removal mechanism by *E. selaginoides*

The mechanisms underlying biosorption are typically complex and depend on the metals used, the type of biomass, and other factors. In the present study, Zn(II) biosorption on *E. selaginoides* is proposed to be controlled primarily by chemisorption *via* surface complexation, as suggested by the obtained isothermal, kinetic, and spectroscopic data.

The predominant chemisorption process is represented by the following Eq. (22), in which the zinc ion forms strong covalent/coordination bonds with two neighbouring carboxylate groups of the alginate chains, which is a feature of the “egg-box” structure found in brown algae.

(22) \begin{eqnarray*}2(\equiv \mathrm{R}-{\mathrm{COO}}^{-}+{\mathrm{Zn}}^{2+})\rightleftharpoons (\equiv \mathrm{R}-\mathrm{COO})_{2}\mathrm{Zn}.\end{eqnarray*}

The good agreement of the experimental zinc adsorption data with the pseudo-second-order model suggests that the rate-determining phase is controlled by the formation of chemical bonds rather than by physisorption or diffusion alone (Ho & McKay, 1999). The mechanism was further confirmed by intra-particle *via* a two-phase mechanism; however, this is not the only rate-controlling phase, as supported by the non-zero intercepts (Fig. S2D; Table 3).

FTIR spectral shifts of certain functional groups, such as amine (–NH), carboxylate (−COO^−^), hydroxyl (–OH), and phosphate (P=O), following adsorption of Zn(II) ions onto the algal biomass suggest their involvement in the binding of zinc ion *via* auxiliary coordination mechanisms. Specifically, the shift of the carboxylate bands is consistent with the possible formation of zinc–carboxylate complexes, which have previously been reported for the alginate-rich cell walls of brown algae (Carreira et al., 2023).

The proposed auxiliary coordination mechanisms are represented by the following Eqs. (23)–(25), and may contribute to zinc biosorption in general but play a supportive role compared to carboxylate-driven complexation.

(23) \begin{eqnarray*}\equiv \mathrm{R}-\mathrm{OH}+{\mathrm{Zn}}^{2+}\rightleftharpoons \equiv \mathrm{R}-\mathrm{O}-{\mathrm{Zn}}^{+}+{\mathrm{H}}^{+}\end{eqnarray*}

(24) \begin{eqnarray*}\equiv R-{\mathrm{NH}}_{2}+{\mathrm{Zn}}^{2+}\rightleftharpoons \equiv \mathrm{R}-{\mathrm{NH}}_{2}\ldots {\mathrm{Zn}}^{2+}\end{eqnarray*}

(25) \begin{eqnarray*}\equiv \mathrm{R}-{\mathrm{PO}}_{4}^{3-}+{\mathrm{Zn}}^{2+}\rightleftharpoons \equiv \mathrm{R}-{\mathrm{PO}}_{4}{\mathrm{Zn}}^{-}.\end{eqnarray*}

Additionally, the possible formation of a bridging zinc–carboxylate complex is illustrated in Eq. (26), which may contribute to the stabilization of the alginate network by bridging two carboxylate groups from distinct polymer chains with a single Zn(II) ion.

(26) \begin{eqnarray*}\equiv \mathrm{R}-{\mathrm{COO}}^{-}+{\mathrm{Zn}}^{2+}+\equiv \mathrm{OOC}-\mathrm{R}\rightleftharpoons \equiv \mathrm{R}-\mathrm{COO}-\mathrm{Zn}-\mathrm{OOC}-\mathrm{R}.\end{eqnarray*}

EDXA analysis also contributes to zinc uptake, revealing the reduction of some cations, such as Na^+^, with the appearance of Zn(II) ion, suggesting partial ion exchange inside the alginate matrix (Reddad et al., 2002). The ion exchange mechanism can be represented by Eq. (27), which illustrates the partial replacement of the Na^+^ cation by Zn(II) ion.

(27) \begin{eqnarray*}2(\equiv \mathrm{R}-{\mathrm{COO}}^{-}{\mathrm{Na}}^{+})+{\mathrm{Zn}}^{2+}\rightleftharpoons (\equiv \mathrm{R}-{\mathrm{COO}}^{-})_{2}\mathrm{Zn}+2{\mathrm{Na}}^{+}.\end{eqnarray*}

Although electrostatic force improves zinc adsorption at neutral pH by decreasing proton competition and encouraging deprotonation from the algal surface, it plays a secondary role compared to chemical adsorption. Equation (28) explains the pH-dependent deprotonation.

(28) \begin{eqnarray*}\equiv \mathrm{R}-\mathrm{COOH}\rightleftharpoons \equiv \mathrm{R}-{\mathrm{COO}}^{-}+{\mathrm{H}}^{+}.\end{eqnarray*}

In general, the biosorption of Zn(II) ion onto *E. selaginoides* occurs primarily through complexation with surface-bound carboxylate groups of alginate, supported by auxiliary coordination with amine, hydroxyl, and phosphate groups, which creates a stable and thermodynamically favorable biosorption system.

### Comparison of marine alga *E. selaginoides* with other biosorbents

According to the results of this study, *E. selaginoides* biomass can be used as an adsorbent for the effective removal of Zn from aqueous solutions. Therefore, the performance of *E. selaginoides* biomass was compared with other adsorbents used for Zn(II) ion removal (Table 5). The maximal biosorption capacity of *E. selaginoides* for Zn(II) ion was close to several microbial biomasses utilized in other earlier investigations. Additionally, the use of the response surface methodology approach results in shorter process times, fewer experiments, and lower material consumption.

**Table 5:** Comparison of marine alga *E. selaginoides* with other biosorbents.

Biosorbent	Maximum biosorption capacity (mg/g)	Zinc concentration (mg/L)	Contact time (min)	pH	Temp. (°C)	Biomass concentration (g/L)	Reference
Immobilized *Chlorella* sp.	28.5	5	6 h	7	25	0.25	Maznah, Al-Fawwaz & Surif (2012)
*Kappaphycus* sp.	16.23	200	90	5	–	4	Rahman & Sathasivam (2015)
*Neopestalotiopsis clavispora*	153	50	30	6	30	–	Hassan et al. (2018)
*Oscillatoria* sp.	54.2	10	24 h	7	28	–	Sezgin, Yildiz & Senturk (2020)
*Ulva lactuca*	23.5	50	120	5	38	1	Ameen et al. (2021)
*Corallina officinalis*	12.8	50	120	5	38	1	Ameen et al. (2021)
*Spirulina platensis*	50.7	60	60	6	25	3	Alharbi et al. (2022)
*Callithamnion corymbosum*	73.69	25	3 h	5	25	2	Ciobanu, Lucaci & Bulgariu (2024)
*Ulva lactuca*	69.29	25	3 h	5	25	2	Ciobanu, Lucaci & Bulgariu (2024)
*Aspergillus flavus*	65.69	500	–	7	25	–	Kumar et al. (2025)
*Ericaria selaginoides*	114.94	50	50	5	25	1	**This study**

## Conclusions

This study affirms that *E. selaginoides* is an effective biosorbent for zinc ions, with a zinc removal efficiency of 87.72 ± 7.3% under optimized conditions obtained by the BBD method. The equilibrium isotherm modeling, kinetic, and surface characterization data show that Zn(II) biosorption on *E. selaginoides* biomass involves a heterogeneous physicochemical process involving interactions with functional groups on the algal biomass, surface complexation, and ion exchange, underscoring *E. selaginoides*’ potential for practical applications in zinc phytoremediation. However, the current investigation was carried out under controlled laboratory conditions using synthetic wastewater, and therefore the performance of *E. selaginoides* under complicated real wastewater conditions still needs to be established. Therefore, future research should assess the long-term performance of the *E. selaginoides* biomass in real wastewater, evaluate biomass regeneration and reusability, and employ advanced spectroscopic techniques such as extended X-ray absorption fine structure (EXAFS) or X-ray photoelectron spectroscopy (XPS) to confirm the proposed biosorption mechanism. These examinations will facilitate the practical application of *E. selaginoides* as a sustainable bio-adsorbent for large-scale wastewater treatment.

## Supplemental Information

10.7717/peerj.21793/supp-1 Supplemental Information 1 Supplemental figures and tables

10.7717/peerj.21793/supp-2 Supplemental Information 2 Biosorption Results

10.7717/peerj.21793/supp-3 Supplemental Information 3 Experimental design

10.7717/peerj.21793/supp-4 Supplemental Information 4 FTIR for untreated algae

10.7717/peerj.21793/supp-5 Supplemental Information 5 FTIR for treated algae

10.7717/peerj.21793/supp-6 Supplemental Information 6 EDX for untreated algae

10.7717/peerj.21793/supp-7 Supplemental Information 7 EDX for treated algae

10.7717/peerj.21793/supp-8 Supplemental Information 8 SEM images

## Additional Information and Declarations

### Data Availability

The following information was supplied regarding data availability: The raw measurements are available in the Supplemental Files.
