# Supplementary material for: Efficient zinc ion biosorption on the biomass of Ericaria selaginoides algae following response surface methodology optimization: uptake behaviors and mechanisms
Source: PeerJ. 2026 Oct 14;14:e21793. doi: 10.7717/peerj.21793 (PMC13676273; doi:10.7717/peerj.21793)
Supplement: Supplementary file 1 [file peerj-14-21793-s006.pdf]

Smp_1080

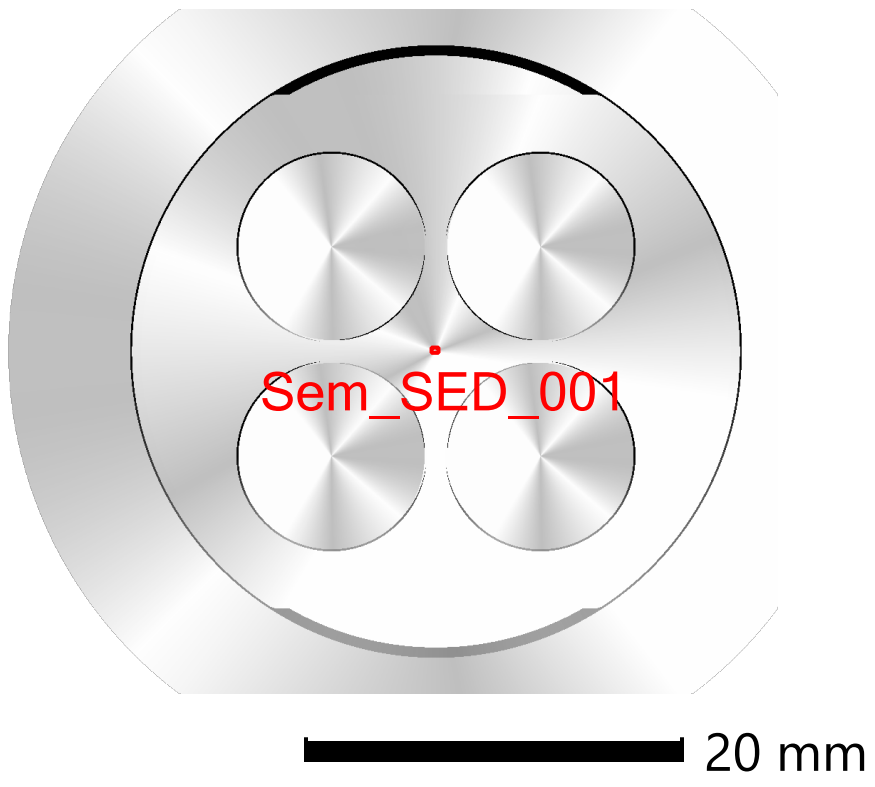

Sem_SED_001

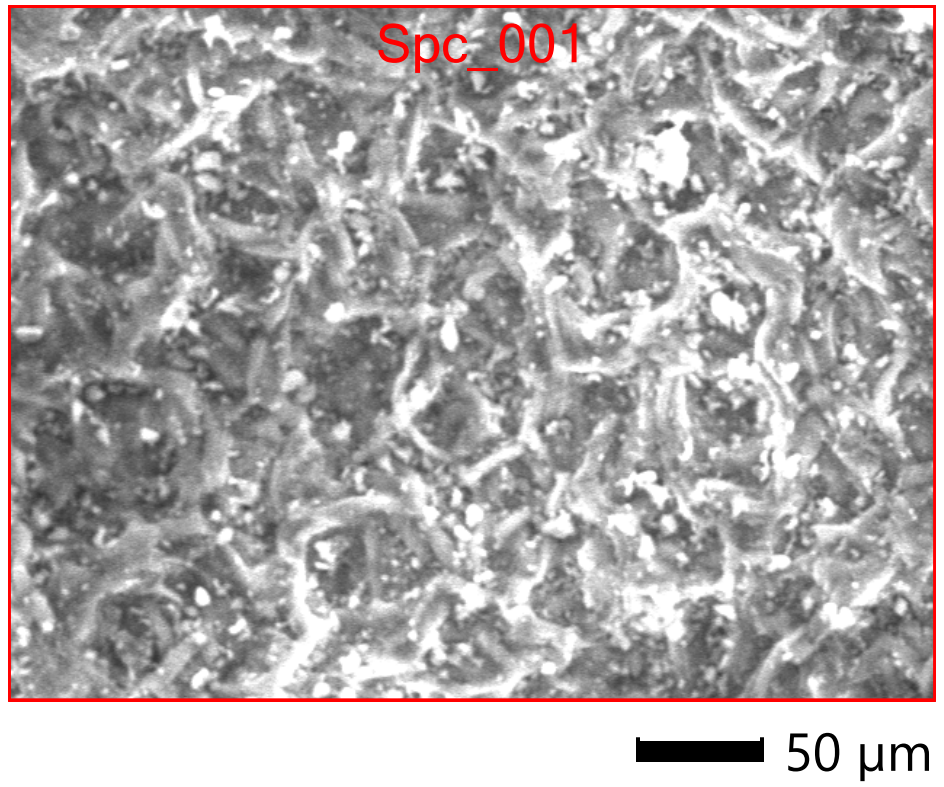

Signal SED
Landing Voltage 30.0 kV
WD 10.1 mm
Magnification x350
Vacuum Mode HighVacuum

Items	Value
measurement conditions	
Acceleration voltage	30.00 kV
Probe current	0.00 nA
Magnification	x 350
Process time	T2
Measurement detector	First
Live time	30.00 seconds
Real time	31.01 seconds
Dead time	3.00
Count rate	6269.00 CPS

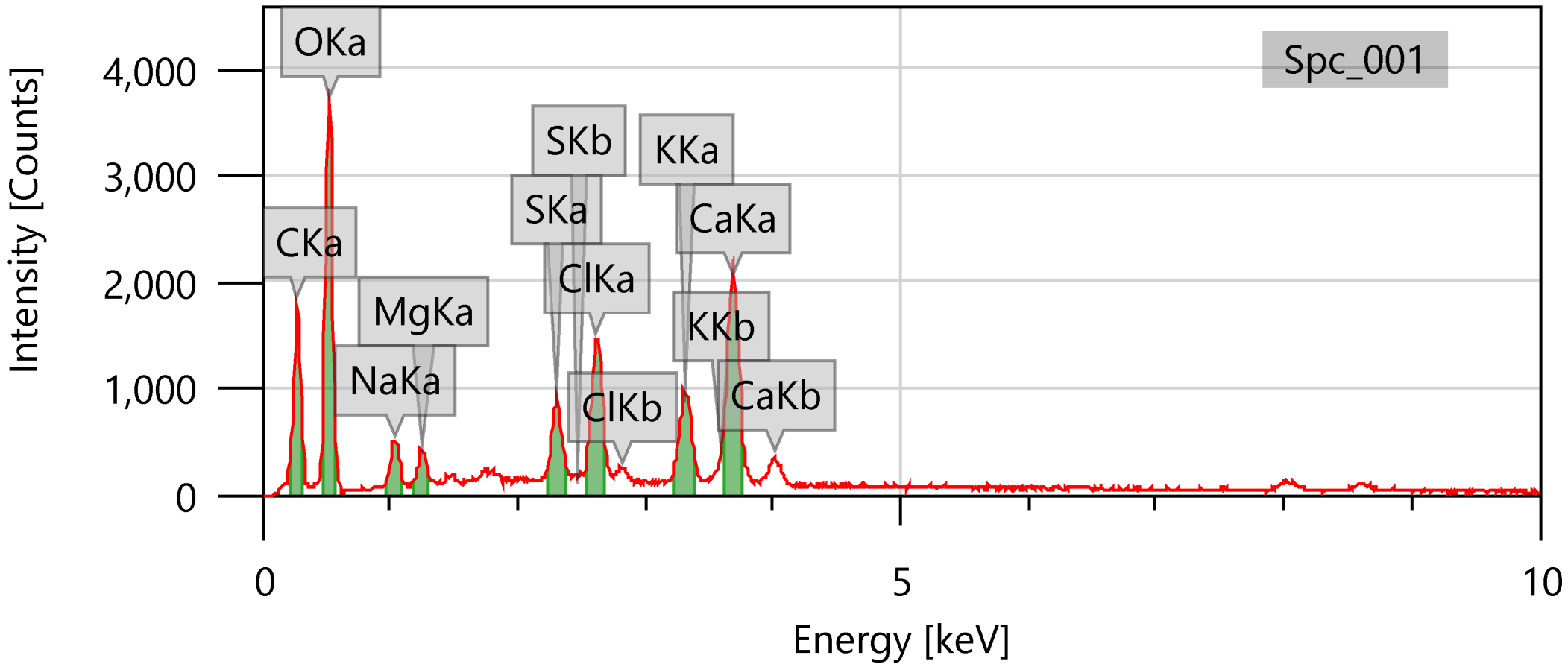

Display name	Standard data	Quantification method	Result Type
Spc_001	Standardless	ZAF	Metal

Element	Line	Mass%	Atom%
C	K	31.71±0.17	40.82±0.22
O	K	54.40±0.35	52.58±0.34
Na	K	2.43±0.06	1.63±0.04
Mg	K	1.07±0.03	0.68±0.02
S	K	1.34±0.02	0.64±0.01
Cl	K	2.63±0.03	1.15±0.01
K	K	1.90±0.03	0.75±0.01
Ca	K	4.54±0.04	1.75±0.02
Total		100.00	100.00
Spc_001			Fitting ratio 0.0313
