# Supplementary material for: Efficient zinc ion biosorption on the biomass of Ericaria selaginoides algae following response surface methodology optimization: uptake behaviors and mechanisms
Source: PeerJ. 2026 Oct 14;14:e21793. doi: 10.7717/peerj.21793 (PMC13676273; doi:10.7717/peerj.21793)
Supplement: Supplementary file 2 [file peerj-14-21793-s007.pdf]

Smp_1080

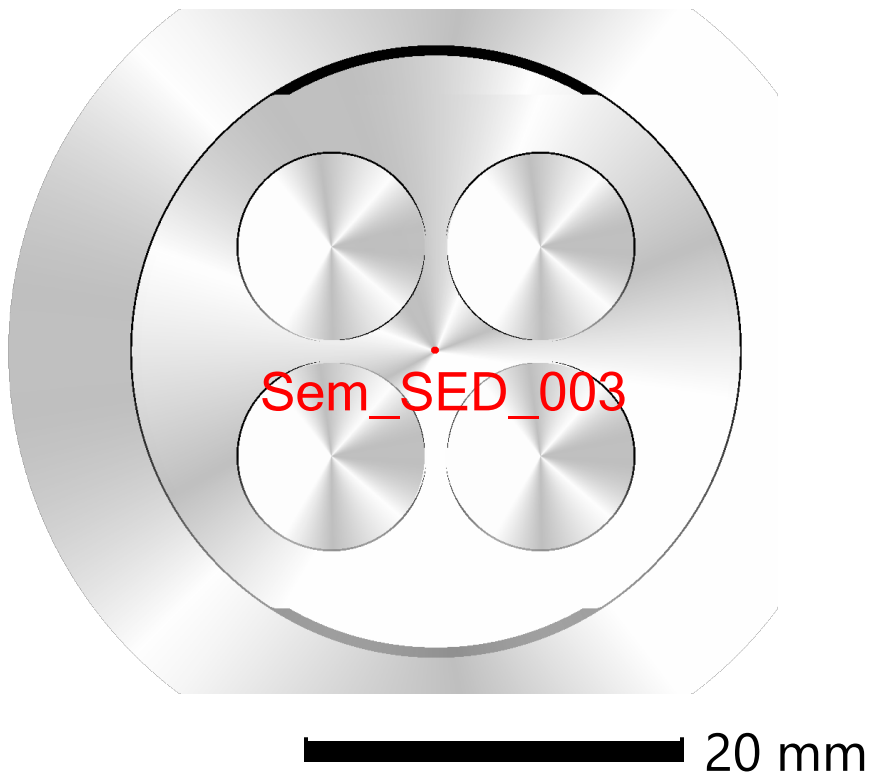

Sem_SED_003

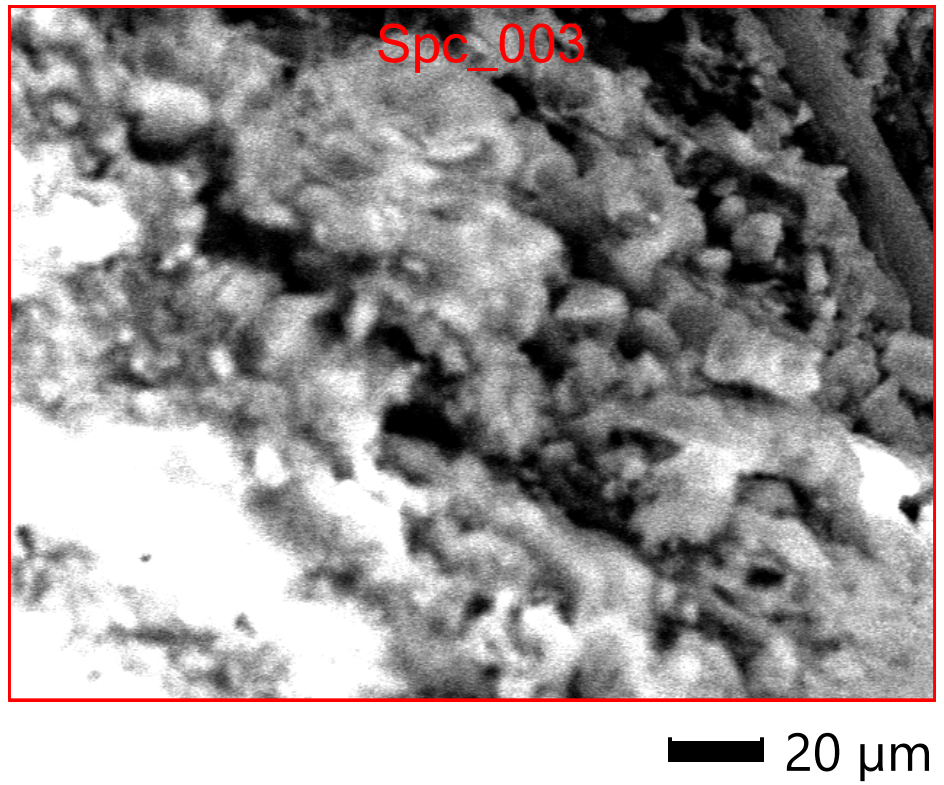

Signal SED
Landing Voltage 30.0 kV
WD 10.1 mm
Magnification x650
Vacuum Mode HighVacuum

Items	Value
measurement conditions	
Acceleration voltage	30.00 kV
Probe current	0.00 nA
Magnification	x 650
Process time	T2
Measurement detector	First
Live time	30.00 seconds
Real time	31.47 seconds
Dead time	5.00
Count rate	12480.00 CPS

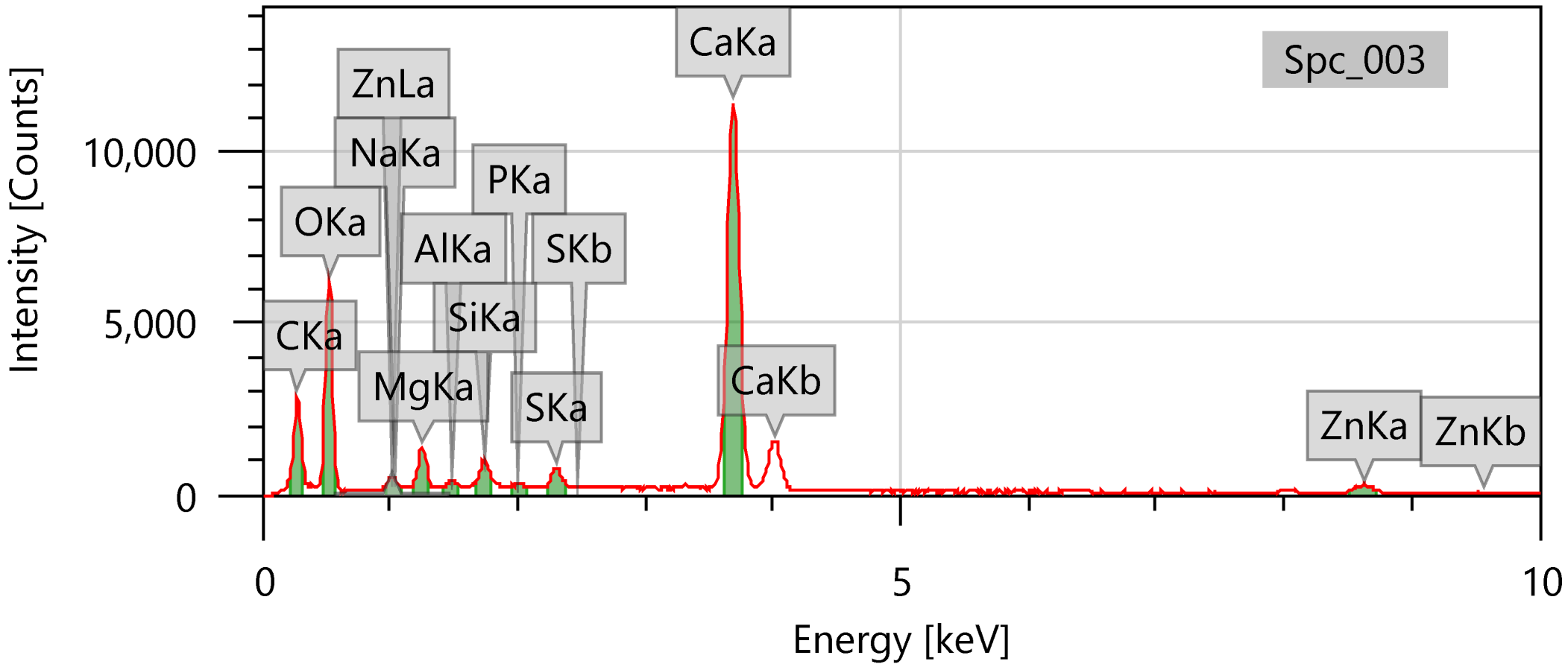

Display name	Standard data	Quantification method	Result Type
Spc_003	Standardless	ZAF	Metal

Element	Line	Mass%	Atom%
C	K	23.23±0.10	32.03±0.14
O	K	56.99±0.28	58.99±0.29
Na	K	0.38±0.03	0.28±0.02
Mg	K	2.16±0.03	1.47±0.02
Al	K	0.28±0.01	0.17±0.01
Si	K	0.79±0.02	0.47±0.01
P	K	0.15±0.01	0.08±0.00
S	K	0.63±0.01	0.32±0.01
Ca	K	14.32±0.05	5.91±0.02
Zn	K	1.07±0.03	0.27±0.01
Total		100.00	100.00
Spc_003			Fitting ratio 0.0121
