# Supplementary material for: Efficient zinc ion biosorption on the biomass of Ericaria selaginoides algae following response surface methodology optimization: uptake behaviors and mechanisms
Source: PeerJ. 2026 Oct 14;14:e21793. doi: 10.7717/peerj.21793 (PMC13676273; doi:10.7717/peerj.21793)
Supplement: Supplementary file 3 [file peerj-14-21793-s008.pdf]

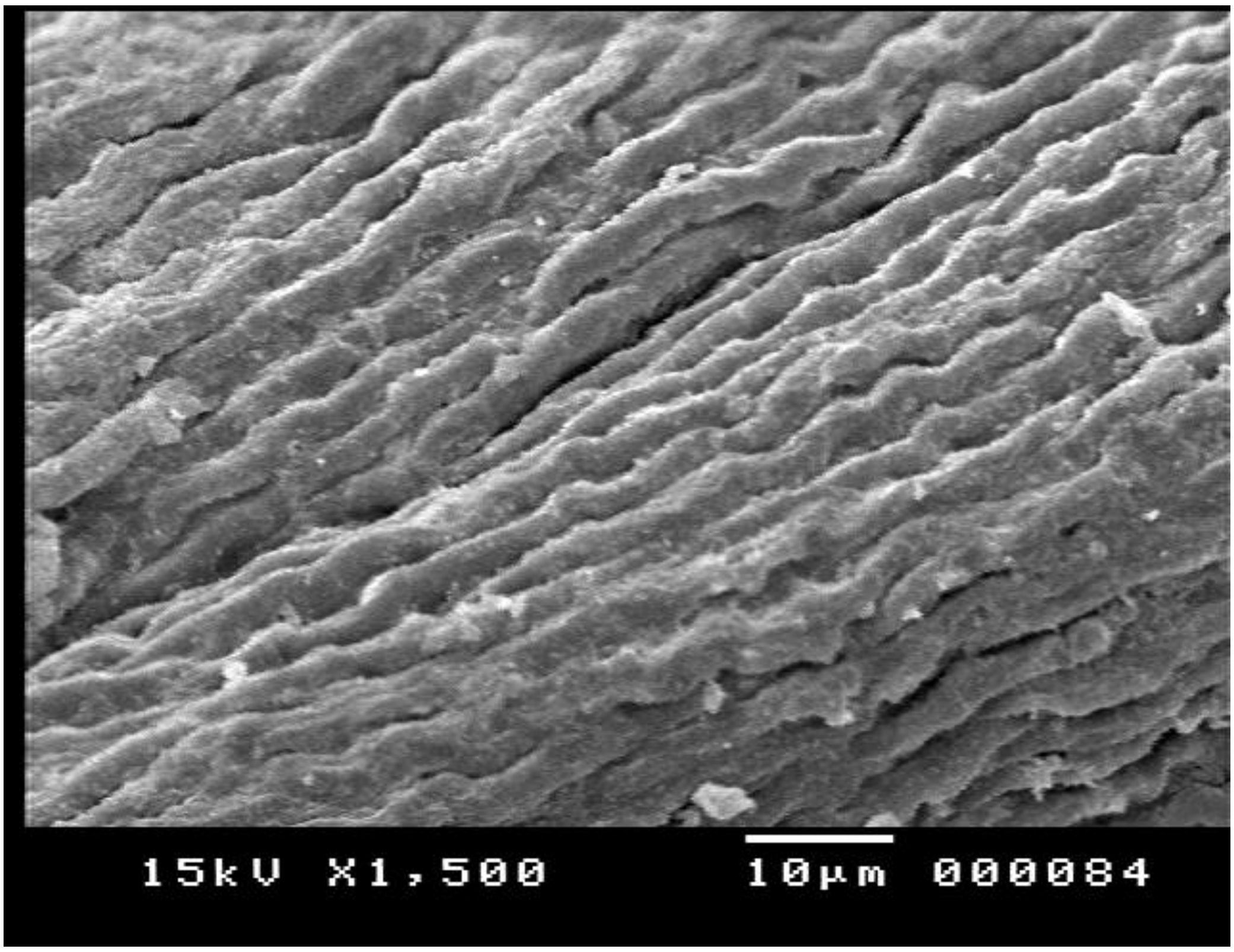

Untreated Algae

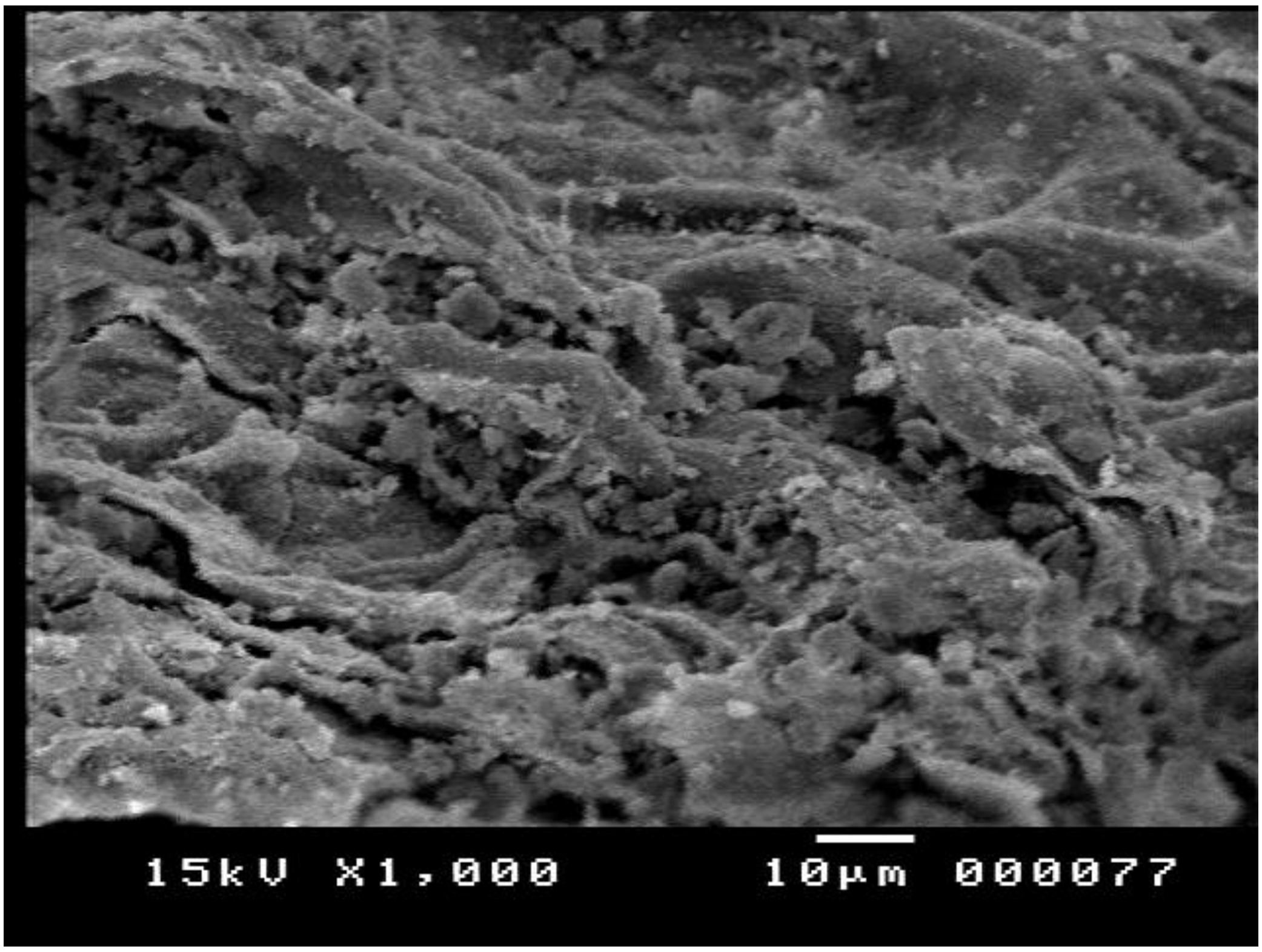

Treated Algae
