# Supplementary material for: Efficient zinc ion biosorption on the biomass of Ericaria selaginoides algae following response surface methodology optimization: uptake behaviors and mechanisms
Source: PeerJ. 2026 Oct 14;14:e21793. doi: 10.7717/peerj.21793 (PMC13676273; doi:10.7717/peerj.21793)
Supplement: Supplementary file 4 [file peerj-14-21793-s001.docx]

**Efficient zinc ion biosorption on the biomass of *Ericaria selaginoides* algae following response surface methodology optimization: uptake behaviors and mechanisms**

Mustafa A. Fawzy^1,2^, Mona Alharbi^3^, Hana Sonbol^3^, Mohamed R. Fouad^4^, Shanoo Sheikh^5^, Sedky H. A. Hassan^6^*

^1^	Botany and Microbiology Department, Faculty of Science, Assiut University, Assiut 71516, Egypt

^2^	Biology Department, College of Sciences, Taif University, P.O. Box 11099, Taif 21944, Saudi Arabia

^3^	Department of Biology, College of Science, Princess Nourah bint Abdulrahman University, P.O. Box 84428, Riyadh 11671, Saudi Arabia

^4^ Department of Pesticide Chemistry and Technology, Faculty of Agriculture, Alexandria University, Aflaton St., 21545, El-Shatby, Alexandria, Egypt

^5^ Department of Health Sciences, College of Health and Rehabilitation Sciences, Princess Nourah bint Abdulrahman University, P.O. Box 84428, Riyadh 11671, Saudi Arabia

^6^	Department of Biology, College of Science, Sultan Qaboos University, Muscat 123, Oman;

*****	Correspondence: [s.hassan@squ.edu.om](mailto:s.hassan@squ.edu.om) (S.H.A.H.); Tel: +96893879120.

Table S1. Box-Behnken experimental design in terms of actual and coded independent parameters of zinc ion.

|  | Actual and coded values | | | Zinc removal (%) | |
| --- | --- | --- | --- | --- | --- |
| Std. order | A: Biosorbent dose (g/100 mL) | B: Biosorbate conc. (mg/L) | C: pH | Actual | Predicted |
| 1 | 0.1(-1) | 30(-1) | 5(0) | 81.33±7.12 | 82.66 |
| 2 | 0.5(+1) | 30(-1) | 5(0) | 78.80±6.87 | 77.67 |
| 3 | 0.1(-1) | 70(+1) | 5(0) | 67.44±6.02 | 68.58 |
| 4 | 0.5(+1) | 70(+1) | 5(0) | 79.99±5.87 | 78.66 |
| 5 | 0.1(-1) | 50(0) | 3(-1) | 76.94±6.43 | 76.05 |
| 6 | 0.5(+1) | 50(0) | 3(-1) | 80.52±7.82 | 82.09 |
| 7 | 0.1(-1) | 50(0) | 7(+1) | 86.20±6.98 | 84.63 |
| 8 | 0.5(+1) | 50(0) | 7(+1) | 82.78±5.98 | 83.67 |
| 9 | 0.3(0) | 30(-1) | 3(-1) | 87.10±6.42 | 86.66 |
| 10 | 0.3(0) | 70(+1) | 3(-1) | 77.93±5.87 | 77.69 |
| 11 | 0.3(0) | 30(-1) | 7(+1) | 89.07±7.32 | 89.31 |
| 12 | 0.3(0) | 70(+1) | 7(+1) | 84.74±8.12 | 85.18 |
| 13 | 0.3(0) | 50(0) | 5(0) | 82.98±7.32 | 83.63 |
| 14 | 0.3(0) | 50(0) | 5(0) | 82.54±6.79 | 83.63 |
| 15 | 0.3(0) | 50(0) | 5(0) | 85.38±5.21 | 83.63 |

A)

| Ceq | Ceq/q |
| --- | --- |
| 1.33 | 0.153403 |
| 4.64 | 0.182965 |
| 8.59 | 0.207438 |
| 16.53 | 0.309145 |
| 21.59 | 0.315597 |

B)

| Ceq | lnqe |
| --- | --- |
| 1.33 | 2.15987 |
| 4.64 | 3.23317 |
| 8.59 | 3.72352 |
| 16.53 | 3.97912 |
| 21.59 | 4.22552 |

C)

| ln Ceq | ln q |
| --- | --- |
| 0.285179 | 2.15987 |
| 1.53471 | 3.23317 |
| 2.1506 | 3.72352 |
| 2.80518 | 3.97912 |
| 3.07223 | 4.22552 |

D)

| ɛ2 | ln q |
| --- | --- |
| 1923214 | 2.15987 |
| 233031 | 3.23317 |
| 74188.7 | 3.72352 |
| 21106 | 3.97912 |
| 12541.3 | 4.22552 |

E)

| ln Ceq | qe |
| --- | --- |
| 0.285179 | 8.67 |
| 1.53471 | 25.36 |
| 2.1506 | 41.41 |
| 2.80518 | 53.47 |
| 3.07223 | 68.41 |

Figure S1. Equilibrium isotherm models, A) Langmuir, B) Jovanovic, C) Freundlich, D) Dubinin and Radushkevich, and E) Temkin.

A)

| Time (min.) | log (qe-qt) |
| --- | --- |
| 0 | 1.36586 |
| 5 | 0.841985 |
| 10 | 0.758912 |
| 15 | 0.673942 |
| 20 | 0.608526 |

B)

| Time (min.) | t/qt |
| --- | --- |
| 0 | 0 |
| 5 | 0.120337 |
| 10 | 0.233863 |
| 15 | 0.342622 |
| 20 | 0.450045 |

**D)**

qt

| t0.5 | qt |
| --- | --- |
| 0 | 25.28 |
| 2.5 | 41.55 |
| 5 | 42.76 |
| 7.5 | 43.78 |
| 10 | 44.44 |

C)

| ln t | qt |
| --- | --- |
| 0 | 25.28 |
| 1.60944 | 41.55 |
| 2.30259 | 42.76 |
| 2.70805 | 43.78 |
| 2.99573 | 44.44 |

Figure S2. Kinetic models, A) pseudo-first-order, B) pseudo-second-order, C) Elovich, and D) Intra-particle diffusion.
